# Molecular analysis of cell-free DNA identifies distinct molecular features in patients with chemosensitive and chemorefractory small cell lung cancer

**DOI:** 10.1186/s40880-019-0363-y

**Published:** 2019-04-18

**Authors:** Jie Zhang, Chen Tian, Fang Lv, Jianfei Wang, Wenbo Han, Jun Nie, Ling Dai, Weiheng Hu, Xiaoling Chen, Xiangjuan Ma, Guangming Tian, Di Wu, Sen Han, Yang Wang, Jieran Long, Ziran Zhang, Jian Fang, Henghui Zhang

**Affiliations:** 1 Department of Thoracic Oncology II, Key Laboratory of Carcinogenesis and Translational Research (Ministry of Education), Peking University Cancer Hospital & Institute, Beijing, 100142 P. R. China; 2 Institute of Infectious Diseases, Beijing Ditan Hospital, Capital Medical University, Beijing, 100011 P. R. China; 3 Beijing Genecast Biotechnology Co., Beijing, 100191 P. R. China

Dear Editor,

Lung cancer is the predominant cause of cancer-related death worldwide [1]. Non-small cell lung cancer accounts for 80%–85% and small cell lung cancer (SCLC) accounts for 15%–20% of all lung cancer cases [2]. Patients with SCLC, a highly aggressive and poorly differentiated malignancy, have an alarming average 5-year overall survival rate of less than 10% [3]. Most patients with SCLC already show clinically detectable metastases at diagnosis and have extremely poor prognoses, even when treated with multimodality therapy [4]. Before the addition of atezolizumab to chemotherapy became the first-line treatment of extensive-stage SCLC, chemotherapy is the primary treatment of SCLC [5]. However, the disease prognosis remains poor because almost all patients relapse after an initial response and eventually die of the disease [5]. Even after robust responses to initial chemotherapy and irradiation, SCLC recurs easily, and no standard therapeutic strategies exist for relapsed SCLC [6]. SCLC causes a high mortality worldwide, but information on the potential molecular mechanism of SCLC is scarce. Therefore, gaining a deep understanding of the molecular pathogenesis of human SCLC is critical for identifying novel potential targets for therapy. In the present study, by using a targeted sequencing approach to detect and compare SCLC driver gene alterations, we identified distinct molecular features in patients with chemosensitive or chemorefractory SCLC.

The details of the patients are shown in Table 1. The study included 28 SCLC patients with a median follow-up duration of 30 months (range, 5–103 months). Twelve patients were classified as chemorefractory because disease progression occurred during first-line therapy or within 90 days of its completion, and 16 patients were defined as chemosensitive because the time to progression after chemotherapy was longer than 90 days. Although the median baseline concentration of cell-free DNA (cfDNA) was relatively lower in the chemosensitive group than in the chemorefractory group, no significant difference was observed between the two groups (*P *> 0.05) (Additional file 1: Figure S1). To investigate the association between the mutation landscape of cfDNA and chemosensitivity, both cfDNA fragments and paired genomic DNA were subjected to enrichment for a 1.15 Mb size panel covering selected exonic regions of 1084 cancer-related genes (Additional file 2: Table S1). Initial analysis of somatic mutations, including single nucleotide variants (SNVs) and short insertions/deletions, showed that alterations in 4 genes were significantly different between the chemosensitive and chemorefractory groups (Fig. 1a, b). The heat-map of the mutation number shows that alterations in adenomatous polyposis coli (*APC*) occurred in 11 (68.8%) patients from the chemosensitive group and in only 2 (16.7%) patients from the chemorefractory group (Fig. 1a, c). Of the 11 patients in the chemosensitive group, 7 (63.6%) had *APC* truncation mutations, whereas the 2 patients in the chemorefractory group had nonsynonymous SNVs (Additional file 3: Figure S2). Mutations in tumor protein 53 (*TP53*), ataxiatelangiectasia mutated (*ATM*), and folliculin (*FLCN*) were commonly detected in the chemorefractory group. We found that the mutation frequencies of *TP53* (66.7% vs. 6.2%), *ATM* (66.7% vs. 12.5%), and *FLCN* (41.7% vs. 0) were all higher in the chemorefractory group than in the chemosensitive group (Fig. 1a, c). Most of the mutations were nonsynonymous SNVs (Additional file 3: Figure S2).

**Table 1:** Characteristics of patients with chemorefractory and chemosensitive small cell lung cancer

Characteristic	Chemorefractory group	Chemosensitive group
Total (cases)	12	16
Age [years; median (range)]	60.5 (47–72)	57 (36–78)
< 40 [cases (%)]	0 (0)	2 (12.5)
40–49 [cases (%)]	1 (8.3)	1 (6. 3)
50–59 [cases (%)]	4 (33.3)	9 (56.3)
60–69 [cases (%)]	5 (41.7)	2 (12.5)
≥ 70 [cases (%)]	2 (16.7)	2 (12.5)
Gender [cases (%)]		
Male	11 (91.7)	9 (56.3)
Female	1 (8.3)	7 (43.8)
Disease stage [cases (%)]		
Limited stage	3 (25.0)	12 (75.0)
Extensive stage	9 (75.0)	4 (25.0)
Cycles of first-line regimen [cases (%)]		
1–2	5 (41.7)	0 (0.0)
3–4	3 (25.0)	5 (31.3)
5–6	4 (33.3)	11 (68.8)
Follow-up [months; median (range)]	10 (5–16)	43.5 (26–103)

**Fig. 1:**
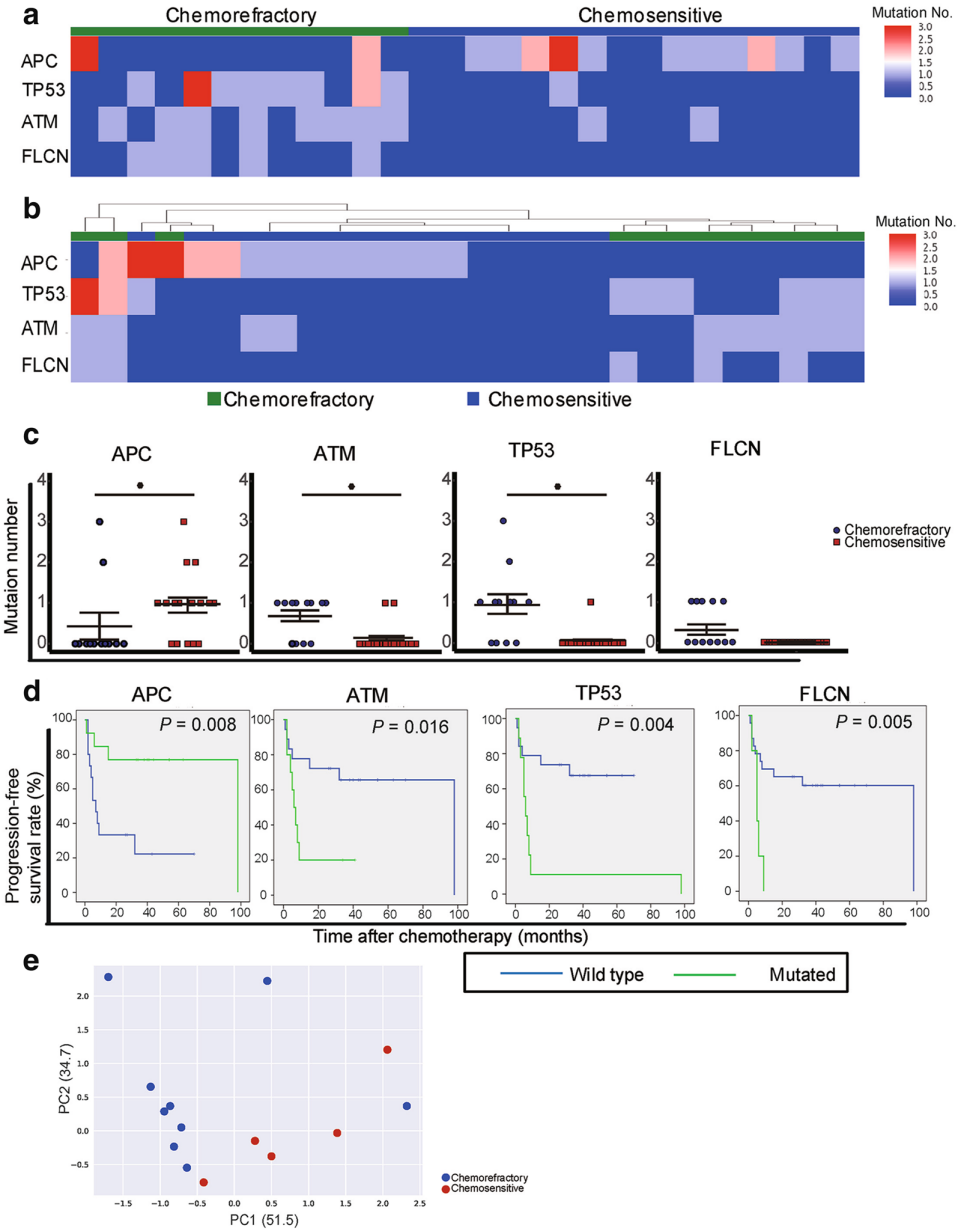
Somatic mutation statuses of genes in plasma cell-free DNA (cfDNA) of chemorefractory and chemosensitive small cell lung cancer (SCLC) patients. **a** The heat-map shows somatic mutation number profiles of adenomatous polyposis coli (*APC*), tumor protein 53 (*TP53*), ataxiatelangiectasia mutated (*ATM*), and folliculin (*FLCN*) identified in plasma cfDNA from each patient. Genes with high mutation numbers are shown in red, and those with low mutation numbers are shown in blue. **b** The heat-map shows hierarchical clustering of the 4 genes which briefly separate chemosensitive (blue) and chemorefractory (green) SCLC patients. **c** Differences in somatic mutation numbers of *APC*, *ATM*, *TP53*, and *FLCN* between the chemorefractory and chemosensitive groups. The data were statistically evaluated with two-tailed *t*-test. The bars indicate standard deviation. *P < 0.05. **d** Kaplan–Meier plots illustrate progression-free survival estimates for SCLC patients with and without mutations of *APC*, *ATM*, *TP53*, and *FLCN*. **e** Principal component analysis (PCA) of somatic mutation number profiles of individual cfDNA from chemosensitive and chemorefractory patients. The percentage variance for each of the principal components is given in parentheses, with separation of chemosensitive and chemorefractory patients seen at the somatic mutation level

To further investigate the prognostic values of *APC*, *ATM*, *TP53*, and *FLCN*, Kaplan–Meier plots were generated. The results showed that *APC* mutations could be considered as a favorable prognostic factor. Patients with *APC* mutations had significantly longer progression-free survival (PFS) than did patients without *APC* mutations (*P* = 0.008) (Fig. 1d). Conversely, the patients with *ATM*, *TP53*, or *FLCN* mutations had significantly shorter PFS than did the patients without such mutations (all *P* < 0.05) (Fig. 1d).

We next performed unsupervised hierarchical clustering of cfDNA based on these 4 genes to explore whether these genes were associated with the chemosensitivity of SCLC. The results demonstrated a clear segregation according to the assigned chemosensitive or chemorefractory status, as expected (Fig. 1b). Unsupervised principal component analysis (PCA) demonstrated a partial separation of samples according to their chemosensitive and chemorefractory statuses (Fig. 1e).

To further explore the relationship between the mutational landscape of cfDNA and the effect of platinum-based chemotherapy, we surveyed the copy number alterations (CNAs) across the genome for all blood samples. Both the genomic DNA of blood cells and cfDNA were extracted to identify meaningful CNA patterns. A heat-map of CNAs from the 28 patients is shown in Fig. 2a. Differential CNAs between the chemosensitive and chemorefractory groups were detected for 5 genes, namely, Src homology 2 domain containing protein 5 (*SH2D5*), carbonic anhydrase 12 (*CA12*), lamin A/C (*LMNA*), patched 1 (*PTCH1*), and DNA ligase 4 (*LIG4*). We next performed unsupervised hierarchical clustering of cfDNA based on these 5 genes to explore whether they were associated with the chemosensitivity of SCLC. Although heterogeneity existed in this profile, chemosensitivity segregation of the patients was not observed (Fig. 2b). Unsupervised PCA based on global chromosomal alterations was conducted, and the results demonstrated a partial separation of the samples according to chemosensitive and chemorefractory statuses (Fig. 2c).

**Fig. 2:**
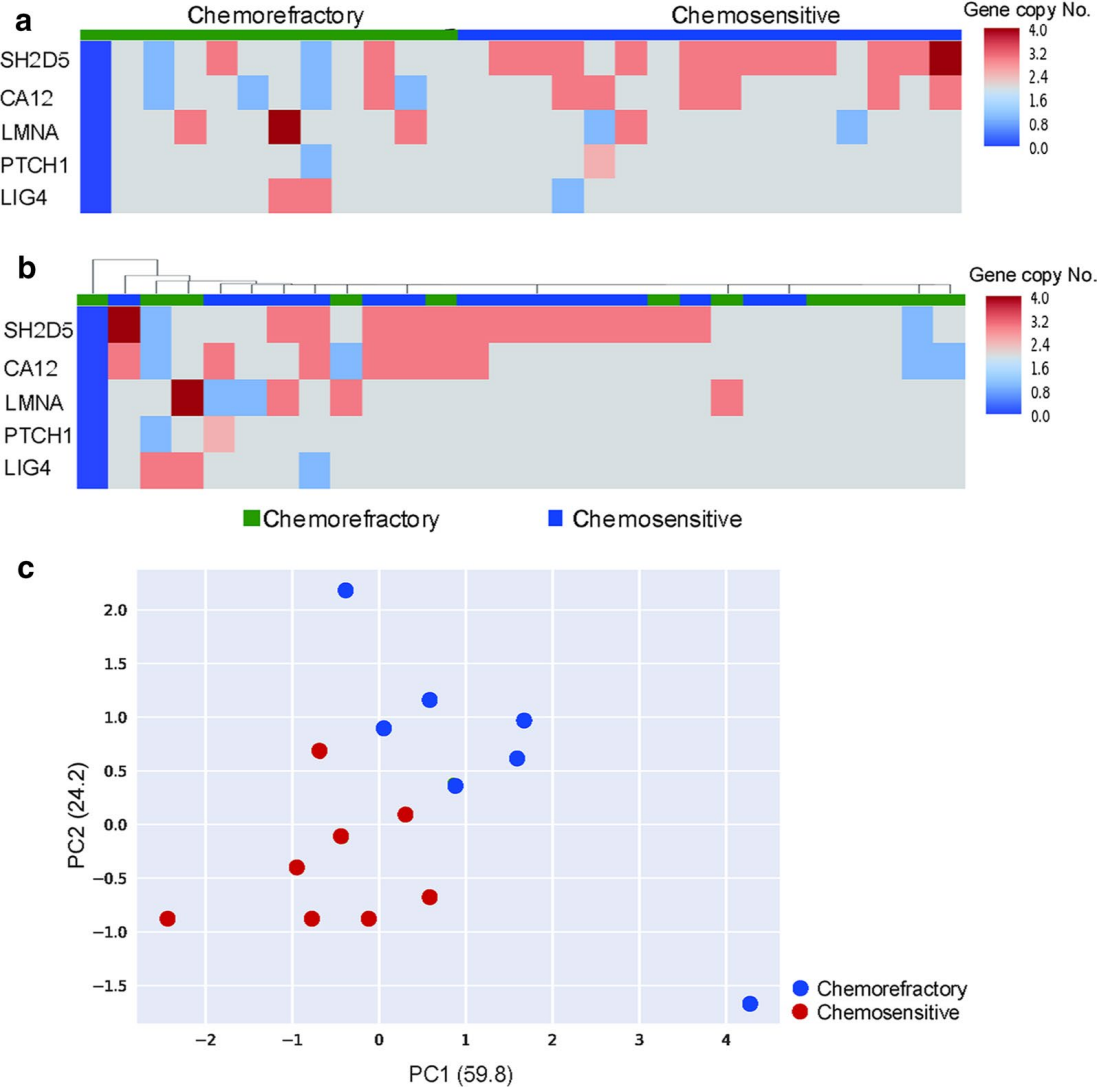
Copy number alterations (CNAs) in plasma cfDNA of chemorefractory and chemosensitive SCLC patients. **a** The heat-map shows CNA profiles of Src homology 2 domain containing protein 5 (*SH2D5*), carbonic anhydrase 12 (*CA12*), Lamin A/C (*LMNA*), Patched 1 (*PTCH1*), and DNA Ligase 4 (*LIG4*) identified in plasma cfDNA of each patient. CNA gains are shown in red, and CNA losses are shown in blue. **b** The heat-map shows hierarchical clustering of the 5 genes which failed to separate chemosensitive (blue) and chemorefractory (green) SCLC patients. **c** PCA of somatic mutation number profiles of individual cfDNA from chemosensitive and chemorefractory patients. The percentage variance for each of the principal components is given in parentheses, with separation of chemosensitive and chemorefractory patients seen at the somatic mutation level

Our results showed that patients with *TP53*, *ATM*, or *FLCN* gene mutations had worse prognoses than did patients with wild-type *TP53*, *ATM*, or *FLCN* genes and that patients with *APC* truncation mutations tended to have tumors being more sensitive to platinum-based chemotherapy.

In 2017, Almodovar et al. [7] reported that mutant allele frequencies and CNAs in the cfDNA of patients with SCLC were associated with the disease burden, depth of the treatment response, and timely warning of disease relapse. In line with these results, we found that the *TP53* mutation was related to the chemotherapeutic response. In addition to *TP53*, we identified other three genes that were associated with chemosensitivity. *ATM* or *FLCN* mutations can be considered as unfavorable prognostic factors for patients with SCLC, whereas patients with *APC* mutations, especially truncation mutations, had longer PFS than did those without *APC* mutations.

In the present study, the CNAs of *SH2D5*, *CA12*, *LMNA*, *PTCH1*, and *LIG4* showed a differential pattern between the chemosensitive and chemorefractory groups. Further large-scale studies and larger panels are needed to validate our findings and explore the association between the cfDNA CNA profile and chemosensitivity to platinum-based chemotherapy for patients with SCLC.

In conclusion, we found that the mutation profile of cfDNA was associated with chemosensitivity to platinum-based chemotherapy in patients with SCLC. Further large-scale prospective studies are needed to validate our findings.

## Additional files

**Additional file 1: Figure S1.** Concentrations of cell-free DNA (cfDNA) in plasma of patients with chemorefractory and chemosensitive small cell lung cancer (SCLC). The concentration of cfDNA was undetectable in 1 patient in chemosensitive group. The bars indicate standard deviation.

**Additional file 2: Table S1.** List of 1084 detected genes in the initial panel (in alphabetical order).

**Additional file 3: Figure S2.** The heat-map shows somatic mutation profiles of adenomatous polyposis coli (*APC*), tumor protein 53 (*TP53*), ataxiatelangiectasia mutated (*ATM*), and folliculin (*FLCN*) identified in plasma cell-free DNA from each patient. SNV, single nucleotide variant.

## Abbreviations

SCLC: small cell lung cancer
PFS: progression-free survival
CNAs: copy number alterations
*APC*: adenomatous polyposis coli
*TP53*: tumor protein 53
*ATM*: ataxiatelangiectasia mutated
FLCN: folliculin
PCA: principal component analysis
*SH2D5*: Src homology 2 domain containing protein 5
*CA12*: carbonic anhydrase 12
*LMNA*: lamin A/C
*PTCH1*: patched 1
*LIG4*: DNA ligase 4
SNVs: single nucleotide variants

## References

[1] Siegel R, Naishadham D, Jemal A (2012). Cancer statistics, 2012. CA Cancer J Clin.

[2] Bernaudin JF (2010). Molecular characteristics of lung cancer. Bull Cancer..

[3] Schneider BJ, Kalemkerian GP (2016). Personalized therapy of small cell lung cancer. Adv Exp Med Biol.

[4] Ehrlich D, Wang B, Lu W, Dowling P, Yuan R (2014). Intratumoral anti-HuD immunotoxin therapy for small cell lung cancer and neuroblastoma. J Hematol Oncol..

[5] Jackman DM, Johnson BE (2005). Small-cell lung cancer. Lancet.

[6] Minami T, Kijima T, Kohmo S, Arase H, Otani Y, Nagatomo I (2013). Overcoming chemoresistance of small-cell lung cancer through stepwise HER2-targeted antibody-dependent cell-mediated cytotoxicity and VEGF-targeted antiangiogenesis. Sci Rep..

[7] Almodovar K, Iams WT, Meador CB, Zhao Z, York S, Horn L (2018). Longitudinal cell-free DNA analysis in patients with small cell lung cancer reveals dynamic insights into treatment efficacy and disease relapse. J Thorac Oncol..

