# Supplementary figures and images for: Molecular analysis of cell-free DNA identifies distinct molecular features in patients with chemosensitive and chemorefractory small cell lung cancer
Source: Cancer Commun (Lond). 2019 Apr 18;39:20. doi: 10.1186/s40880-019-0363-y (PMC6472086; doi:10.1186/s40880-019-0363-y)

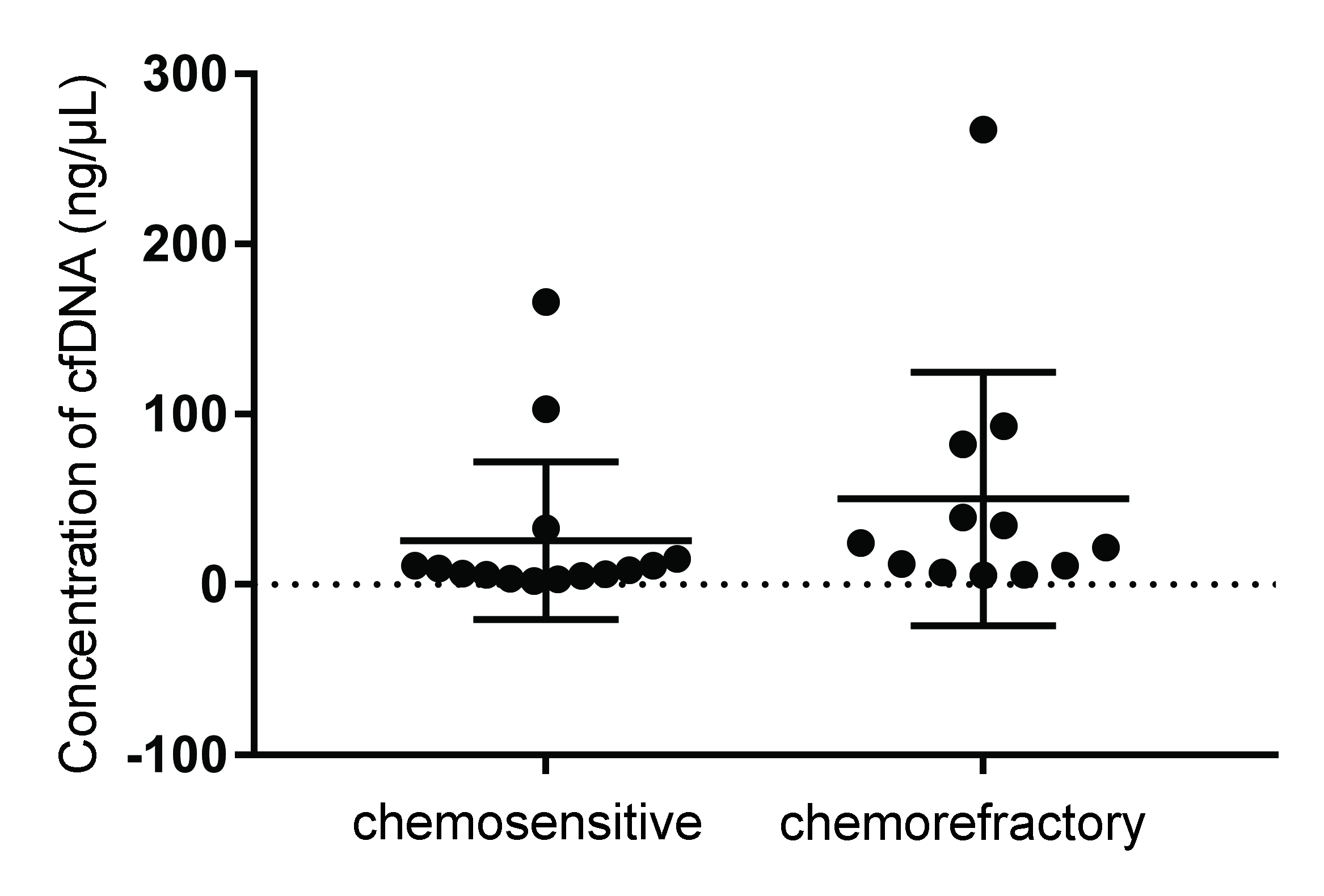

Supplement: Supplementary file 1 — Additional file 1: Figure S1. Concentrations of cell-free DNA (cfDNA) in plasma of patients with chemorefractory and chemosensitive small cell lung cancer (SCLC). The concentration of cfDNA was undetectable in 1 patient in chemosensitive group. The bars indicate standard deviation. [file 40880_2019_363_MOESM1_ESM.tiff]

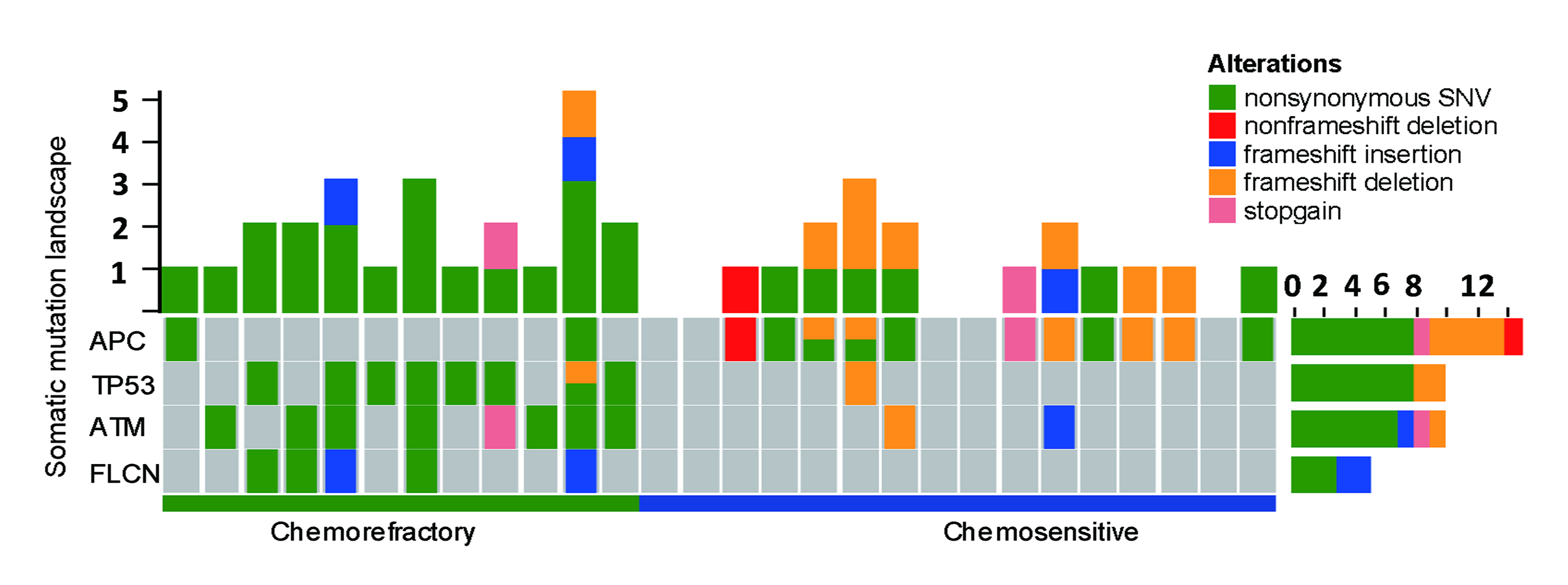

Supplement: Supplementary file 3 — Additional file 3: Figure S2. The heat-map shows somatic mutation profiles of adenomatous polyposis coli (APC), tumor protein 53 (TP53), ataxiatelangiectasia mutated (ATM), and folliculin (FLCN) identified in plasma cell-free DNA from each patient. SNV, single nucleotide variant. [file 40880_2019_363_MOESM3_ESM.tiff]
